# Transactions of the Texas State Medical Association, 1885

**Published:** 1886-01

**Authors:** 


TRANSACTIONS OF THE TEXAS STATE MEDICAL ASSOCIATION. Seventeenth Annual Session, held at Houston, Texas, April 21, 22, 23, 1885. Austin, Texas: Printed for the Texas State Medical Association. 1885.

The correspondence of this volume in all its material characteristics to the tasteful and artistic appearance of the Transactions of the Medical Association of Georgia, for past years, indicates an appreciation on the part of our Texas brethren of the Georgia model which should gratify every member of our body. With the lone star in the place of the clasped hands, and the black instead of the green cover, there is scarcely another feature by which to distinguish the Texas from the Georgia volume of Transactions, either in their exterior aspect or the superb execution of the printing upon a superior quality of paper, which, with a broad margin to the fine octavo page, recommend these records to the reader. In addition to the name, post-office, county and year of admission, the roll of members includes the age, nativity, when and where graduated; but there are many vacancies in those last records, from the neglect of members in supplying them dates. The contributions are numerous and varied, filling three hundred and forty-five pages of the four hundred and thirty that make up the volume.

For the most part the subjects presented are the outgrowth of experience, and are treated from a practical standpoint, so as to be especially useful to the general practitioner. Those manifesting least familiarity with the bibliography of the matters under consideration are still instructive by the detail of symptoms and plain statement of facts. A case of dilatation of stricture of the œsophagus, with the inflatable dilatation, by Dr. C. W. Trueheart, of Galveston, in a child two and a half years old, is of special interest. The address of the President, Dr. H. C. Ghent; a paper on the influence of climate, by Dr. S. H. Stout, and the report on surgery, by Dr. E. J. Beall, are deserving of careful consideration.

